# Supplementary material for: Retrieving high-resolution information from disordered 2D crystals by single-particle cryo-EM
Source: Nat Commun. 2019 Apr 12;10:1722. doi: 10.1038/s41467-019-09661-5 (PMC6461647; doi:10.1038/s41467-019-09661-5)
Supplement: Supplementary file 4 — Description of Additional Supplementary Files [file 41467_2019_9661_MOESM4_ESM.docx]

**Description of Additional Supplementary Files**

**Supplementary Movie 1**. Blinking of the CNBD. A simple morph from model #4 (“compact” conformation) to model #1 (“extended” conformation) in the ensemble derived from the 3D classes. Each chain is shown with a different ribbon color. Potassium ions are colored purple, and the side chains in the selectivity filter are explicitly shown (residues 175-178). a) Side view; b) CNBD view; c) pore view. Movie generated in UCSF Chimera.

**Supplementary Movie 2**. Blinking of the CNBD and tilting of the VSD. A simple morph from model #6 (“compact” conformation) to model #1 (“extended” conformation) in the ensemble derived from the 3D classes. Each chain is shown with a different ribbon color. Potassium ions are colored purple, and the side chains in the selectivity filter are explicitly shown (residues 175-178). a) Side view; b) CNBD view; c) pore view. Movie generated in UCSF Chimera.

**Supplementary Movie 3.** Rotation of the CNBD and the selectivity filter with respect to the TMD. A simple morph from model #5 (intermediate “compact” conformation) to model #3 (intermediate “extended” conformation) in the ensemble derived from the 3D classes. Each chain is shown with a different ribbon color. Potassium ions are colored purple, and the side chains in the selectivity filter are explicitly shown (residues 175-178). a) Side view; b) CNBD view; c) pore view. Movie generated in UCSF Chimera.
